# Association between active pulmonary tuberculosis and circulating microRNAs: a preliminary study from Turkey

**DOI:** 10.3906/sag-2004-307

**Published:** 2021-08-30

**Authors:** Fatma KALAYCI YÜKSEK, Cenk KIĞ, Gönenç ORTAKÖYLÜ, Meltem UZUN

**Affiliations:** 1 Department of Medical Microbiology, Faculty of Medicine, İstanbul University, İstanbul Turkey; 2 Department of Medical Microbiology, Faculty of Medicine, İstanbul Yeni Yüzyıl University, İstanbul Turkey; 3 Department of Medical Biology and Genetics, Faculty of Medicine, İstanbul Yeni Yüzyıl University, İstanbul Turkey; 4 Department of Pulmonology, Yedikule Chest Diseases and Thoracic Surgery Training and Research Hospital, İstanbul Turkey

**Keywords:** Circulating-microRNAs, active pulmonary tuberculosis, biomarker, *Mycobacteriumtuberculosis*

## Abstract

**Background/aim:**

Tuberculosis is a public health problem that still remains significant. For prevention, diagnosis, and treatment of tuberculosis more effective novel biomarkers are needed. MicroRNAs can regulate innate and adaptive immune responses, alter host-pathogen interactions, and affect progression of diseases. The relationship between microRNA expression and active pulmonary tuberculosis (APT) has not yet been investigated in the Turkish population. We aimed to test the potential diagnostic value of some microRNAs whose levels were previously reported to be altered in APT patients.

**Materials and methods:**

Using two different references (U6 and miR-93), we compared the expression levels of potentially important microRNAs in serum of APT patients with healthy individuals using quantitative polymerase chain reaction (qPCR).

**Results:**

miR-144 expression level was down-regulated in APT patients when either U6 or miR-93 was used for normalization. When data was normalized with miR-93, a statistically significant decrease in miR-125b (0.8 fold) and miR-146a (0.7 fold) expression levels were observed, while no differences were detected for U6. The receiver operating characteristic suggested that miR-144 may be a candidate biomarker for discriminating APT patients and controls (p < 0.05) both for U6 and miR-93.

**Conclusion:**

These findings suggest that miR-144 can have potential as a biomarker for APT. Using a single reference may be misleading in evaluation of microRNA expression. U6 and miR-93 can be used in combination as references for normalization of serum microRNA expression data.

## 1. Introduction

*Mycobacterium* *tuberculosis* (Mtb), the causative agent of tuberculosis, is estimated to be responsible for ten million new cases of tuberculosis (TB) and approximately 1.5 million deaths in 2018 throughout the world. Tuberculosis is still one of the top 10 causes of death worldwide^1^World Health Organization (2019). Global Tuberculosis Report [online]. Website https://www.who.int/tb/publications/global_report/en/ [accessed 21 July 2020].. Early diagnosis of TB infection is important to prevent the spread of disease and for its’ treatment. In addition to the PCR-based molecular tests such as GenXpert (Cepheid), Mtb culture is currently accepted as the gold standard for TB diagnosis. Mtb isolation is time-consuming and microscopic analysis can yield subjective results. However, sensitive molecular diagnostic techniques such as PCR are only available in well-equipped laboratories. Due to the limited access to molecular diagnostic tools, simpler methods that allow for detection of stably expressed novel biomarkers are required [1–3]. 

In recent years, microRNAs (miRNAs) have been investigated for their potential role in the area of TB diagnosis. MicroRNAs are small noncoding RNA molecules which are known to modulate in RNA interference and which affect to protein translation or decay mRNA. It is known that miRNAs effect epigenetic mechanisms affecting by DNA methyltransferases, histone deacetylases, and histone methyltransferases. They also play roles in DNA methylation, RNA, and histone modifications. The interactions of miRNAs and epigenetic mechanisms are reported as very important for modulating cell proliferation, apoptosis, differentiation, and regulation of immune response mechanisms [4–6]. Therefore these molecules are shown to be involved in modulating various biological and pathological processes such as inflammation, cancer, and infectious diseases [7–9]. More importantly, miRNAs are found to be stable in serum and plasma samples which are suitable for long-term storage. In the last decade, a number of researchers have reported that changes in the miRNA expression profiles in bodily fluids can reflect TB pathologies and immunological modulation [6,10–34]. 

Despite their potential in TB diagnosis, association between miRNA levels and TB infection has not been investigated in Turkish patients to date. In this respect, we aimed to compare the changes in the serum miRNA levels between TB patients and the control group. Eleven miRNAs had been selected to be tested for their diagnostic potential based on a literature survey analysis. These miRNAs were reported to show altered expression during TB infection as well as being involved in the regulation of inflammatory responses against TB [12–34]. The roles of these miRNAs have been summarized in Table 1 [35–38]. 

**Table 1:** MiRNAs associated with tuberculosis and their roles in immunity against TB.

MiRNAs	The roles
miR-29a	The inhibition of interferon-γ
miR-21	The suppression of IL-1β and increasing of IL-10
miR-146a	Induction of intracellular growth of bacilli, the inhibition of TNF-α, IL-1β, and IL-6
miR-223	The suppression of apoptosis and inhibition of IL-6
miR-142	The suppression of M. tuberculosis phagocytosis
miR-125b	The reduction of inflammatory responses via TNF-α
miR-144	Inhibition of interferon- γ ,TNF-α, and T cell proliferation
miR-155	Promotes Th17 differentiation and Interferon-γ production in CD4+ and CD8+ T cells and affecting of apoptosis
miR-99	The suppression of pro-inflammatory cytokine production in dendritic cells
miR-361-5p	Targets SP-1 transcription factor (SP1) that is defined as a key signaling pathway for IL-10 expression in the lung
miR-582-5p	The inhibition of monocyte apoptosis

It is well known that normalization of data against a reliable reference is the most critical step of RT-qPCR experiments. Considering that some researchers reported variations in the most commonly used U6 reference [39,40], we also aimed to include a second alternative reference. Some researchers suggested alternative references for normalization in TB studies such as miR-16 [12], MammU6, RNU44, and RNU48 [16], let-7 [41]. However, as U6 has been used in majority of studies [14,17,23,25], in this study U6 is preferred for normalization. Moreover, Barry et al. reported that significant variability in miRNA levels across Australian and Chinese populations, independent of disease status. They showed that miR-93 was a suitable reference for normalizing miRNA in plasma of TB patients [42]. Therefore, we used more than one reference to be able to provide more reliable data.

## 2. Materials and methods

### 2.1. Study design

We performed a literature survey by reviewing 23 articles which investigated the changes in miRNA expression profiles in TB patients from around the world (Table 2) [12–34]. Among the differentially expressed miRNAs, the most frequently reported and clinically confirmed ones were selected such as miR-29, miR-21, miR-146a, miR-223, miR-142, miR-125b, miR-144, miR-155, miR-99, miR-361, and miR-582-5p (Table 2) for testing in a study group consisting of 20 TB patients and 20 healthy controls from Turkey.

**Table 2:** MiRNAs associated with tuberculosis in our literature review.

MiRNAs	Serum	Plasma	Sputum	PBMC*	PFMC*	CSF*	T Cells	Whole blood	Neutrophil	Monocytes and macrophages
miR-29a	Fu et al., 2011 [14] Draz et al., 2014 [15]Qi et al., 2012 [12]	Barry et al.,2018 [18]	Fu et al., 2011 [14]	Pan et al.,2017 [20]Spinelli et al.,2013 [21]		Pan et al.,2017 [20]	Kleinsteuber et al., 2013 [26]Fu et al., 2014 [27]	Latorre et al.,2015 [28]		
miR-21		Barry et al.,2018 [18]		Xu ve ark., 2013 [22]Wang et al., 2011 [23]Wu et al., 2014 [24]			Kleinsteuber et al., 2013 [26]			
miR-146a	Fu et al., 2011 [14] Miotto et al., 2013 [16]	Barry et al.,2018 [18]		Spinelli et al.,2013 [21]	Spinelli et al., 2013 [21]					
miR-223				Wang et al., 2011 [23]Spinelli et al., 2013 [21]	Spinelli et al., 2013 [21]					Xi et al.,2015 [29]
miR-142				Spinelli et al.,2013 [21]			Kleinsteuber et al., 2013 [26]			
miR-125b	Fu et al., 2011 [14]Wang et al., 2016 [33]		Yi et al., 2012 [31]					Zhou et al.,2016 [13]		
miR-144	Lv et al., 2016 [17]	Ndzi et al.,2019 [19]	Lv et al., 2016 [17]	Liu et al.,2011 [25]	Spinelli et al., 2013 [21]			Zhou et al.,2016 [13]		
miR-155	Fu et al., 2011 [14]	Ndzi et al.,2019 [19]		Spinelli et al.,2013 [21]						Huang et al.,2015 [30]
miR-99b		Barry et al.,2018 [18]	Yi et al., 2012 [31]						van Rensburget al., 2018 [32]	
miR-361-5p	Qi et al., 2012 [12]Draz et al., 2014 [15]	Ndzi et al.,2019 [19]						Latorre et al.,2015 [28]		
miR-582-5p	Fu et al., 2011 [14]Yi et al., 2012 [31]									Liu et al.,2013 [34]

*PBMC: peripheral blood mononuclear cells, PFMC: pleura fluid mononuclear cells, CSF: cerebrospinal fluid.

### 2.2. Ethics statement

This study was performed in accordance with guidelines of the Ethics Committee of Yedikule Chest Diseases and Thoracic Surgery Training and Research Hospital, Istanbul (Approval 2016/60). Informed consent was obtained from all participants prior to beginning the study. 

### 2.3. Human subjects 

A total of 40 participants were recruited in this study. Twenty patients with active pulmonary TB who presented to “Yedikule Chest Diseases and Thoracic Surgery Training and Research Hospital, Istanbul” between September 2017 and May 2018 were included in the study [5 females and 15 males; the median age of active pulmonary TB patients was 34.50 (22.50–38.50)]. Eligibility for patients’ entry into the study included the presence of the typical symptoms for pulmonary TB and *M. tuberculosis* positive culture test results. Patients who had diabetes, cancer, and other pulmonary diseases or co-infection with other pathogens and/or receiving antituberculosis treatment were excluded [14,27,31]. Twenty healthy subjects (15 males and 5 females) free of active or latent TB infection and who did not display any clinical symptoms of any other infectious or noninfectious diseases were recruited as controls [the median age of controls was 30 (28–35)]. A questionnaire was verbally administered to healthy controls and those who declared not having a history of tuberculosis were included in this study. Prior exposure of healthy individuals to TB was not further investigated by using QuantiFERON-TB Gold (QFT) and/or purified protein derivative (PPD) tests. None of the participants had received any drugs at the time of blood collection. No significant difference was found between the two groups, with respect to gender, age, and smoking distribution (Table 3). 

**Table 3:** Characteristics of participants.

		Patient group(n = 20)	Healthy control group(n = 20)	p-value
Age (years)	Median [first IQ-third IQ]	34.50 [22.50–38.50]	30 [28–35]	0.758a
Sex (%)	Female	5 (25%)	5 (25%)	>0.99b
	Male	15 (75%)	15 (75%)	
Smoker (%)	Positive	16 (80%)	17 (85%)	>0.99c
	Negative	4 (20%)	3 (15%)	
Sex (%)	Female	5 (25%)	5 (25%)	>0.99b
	Male	15 (75%)	15 (75%)	
Smoker (%)	Positive	16 (80%)	17 (85%)	>0.99c
	Negative	4 (20%)	3 (15%)	

a: Mann–Whitney U test,b: Pearson Chi-Square test, c: Fisher’s Exact test.

### 2.4. RNA extraction and quantitative PCR 

Blood sample (~5 mL) was drawn into a sterile polyolefin resin tube without anticoagulant for RNA isolation. Serum separation and RNA extraction were performed within 2 h after blood collection. RNA extraction from serum samples was performed using the miRCURY RNA isolation kit—Biofluids (Exiqon, Denmark) according to manufacturer’s instructions. RNA quality and concentration were determined with a NanoDrop 2000 spectrophotometer Thermo Scientific (Waltham. USA). Reverse transcription was carried out using miRCURY LNAUniversal TR microRNA PCR kit (Exiqon, Denmark) according to the manufacturer’s instructions and reactions were performed in a thermal cycler (42 °C for 60 min. and 95 °C for 5 min). After reverse transcription step, cDNAs were diluted to 1/40. 

Quantitative PCR reactions were carried out using miRCURY LNA Universal RT microRNA PCR kit in a BIORAD instrument (Bio-Rad, Hercules, CA, USA) according to the instructions of the manufacturer. PCR conditions were as follows: 95 °C for 10 min, followed by 40 cycles at 95 °C for 10 s and 60 °C for 1 min. Total PCR volume was 10 µL. Normalization was performed separately using both U6 small nuclear RNA level and miR-93 levels. Ct values over 40 were not considered significant. The experiments were conducted in duplicates and the results were presented as fold change for each miRNA. Calculations were made using the 2^–ΔΔCt^ method. Sensitivity and accuracy of the protocol were tested at different levels by performing melting curve analysis, using spike RNA as control or amplification curves of serial dilutions for the target miRNAs. Primer sequences can be provided upon request. 

### 2.5. Statistical analysis

In order to calculate the sample size, priori power analysis was performed. Power analysis was performed under G*Power 3.1^2^G*Power (2020). [online]. Website http://www.gpower.hhu.de/ [accessed 21 July 2020].. Shapiro–Wilk test was used for assessing whether the variables follow normal distribution or not. According to the normality test results, Mann–Whitney U test was used in comparison between two groups. Pearson Chi-square test and Fisher’s exact test were used for comparing categorical variables. In order to evaluate the diagnostic accuracy, we used receiver operating characteristic (ROC) curve analysis. SPSS (IBM Corp. Released 2012. IBM SPSS Statistics for Windows, v: 21.0, Armonk, NY: IBM Corp.) and MedCalc v: 12.3.0.0 were used for statistical analysis and a p-value < 0.05 was considered statistically significant. The area under the curve (AUC) and 95% confidence intervals (CI) were calculated for specificity and sensitivity. For ROC analysis we applied the generally accepted criteria, values between 90%–100% are accepted excellent, 80%–90% good, 70%–80% fair, 60%–70% poor, and 50%–60% bad (or failed) in general. 

## 3. Results

Based on our literature survey (Table 2) we have selected 11 miRNAs for evaluation of their diagnostic potential in TB patients from Turkey. For this purpose, we compared the expression levels of these target miRNAs between patients and the control group via qPCR as explained under the materials and methods section. 

**Figure 1:**
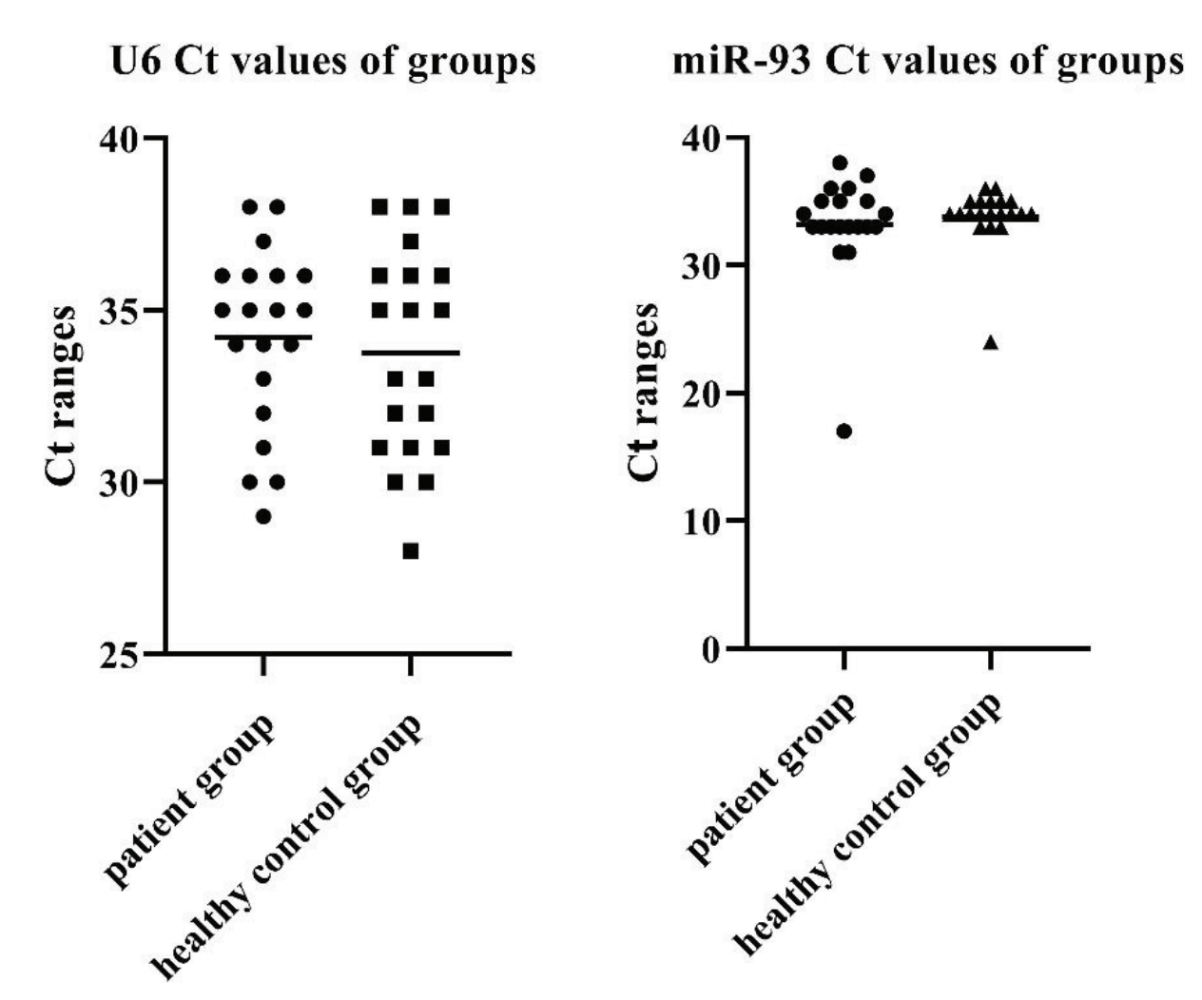
The Ct values of U6 and miR-93 in patient and healthy control groups.

Although no reliable reference has been proposed for normalization of circulating miRNAs, U6 and miR-93 were previously used as endogenous controls due to their relatively stable expression levels in TB patients [14,18,31,42]. Thus, both U6 and miR-93 references for normalization of miRNA expression data were included in this study. The Ct values for U6 and miR-93 examined in tuberculosis and healthy control groups were shown in Figure 1.

In agreement with previously reported data, it was found that the expression levels of miR-29, miR-21, miR-146a, miR-223, miR-142, miR-125b, miR-155, miR-99, miR-361, and miR-582-5p were altered in TB patients as seen in Table 4. However, only the changes in miR-144 expression level (–0.8 fold, p < 0.05) were found to be statistically significant when data was normalized against U6 expression level (Figures 2a and 2b). 

**Table 4:** Fold change distributions in miRNA expression levels when data normalized against U6 RNA *(p: 0.017).

miRNAs	Fold changes in patients with active pulmonary TB compared to healthy controls
miR-29a	–0.52
miR-21	–0.20
miR-146a	–0.61
miR-223	0.13
miR-142	–0.19
miR-125b	–0.69
miR-144	–0.86*
miR-155	–0.13
miR-99b	–0.24
miR-361-5p	0.20
miR-582-5p	–0.13

**Figure 2a:**
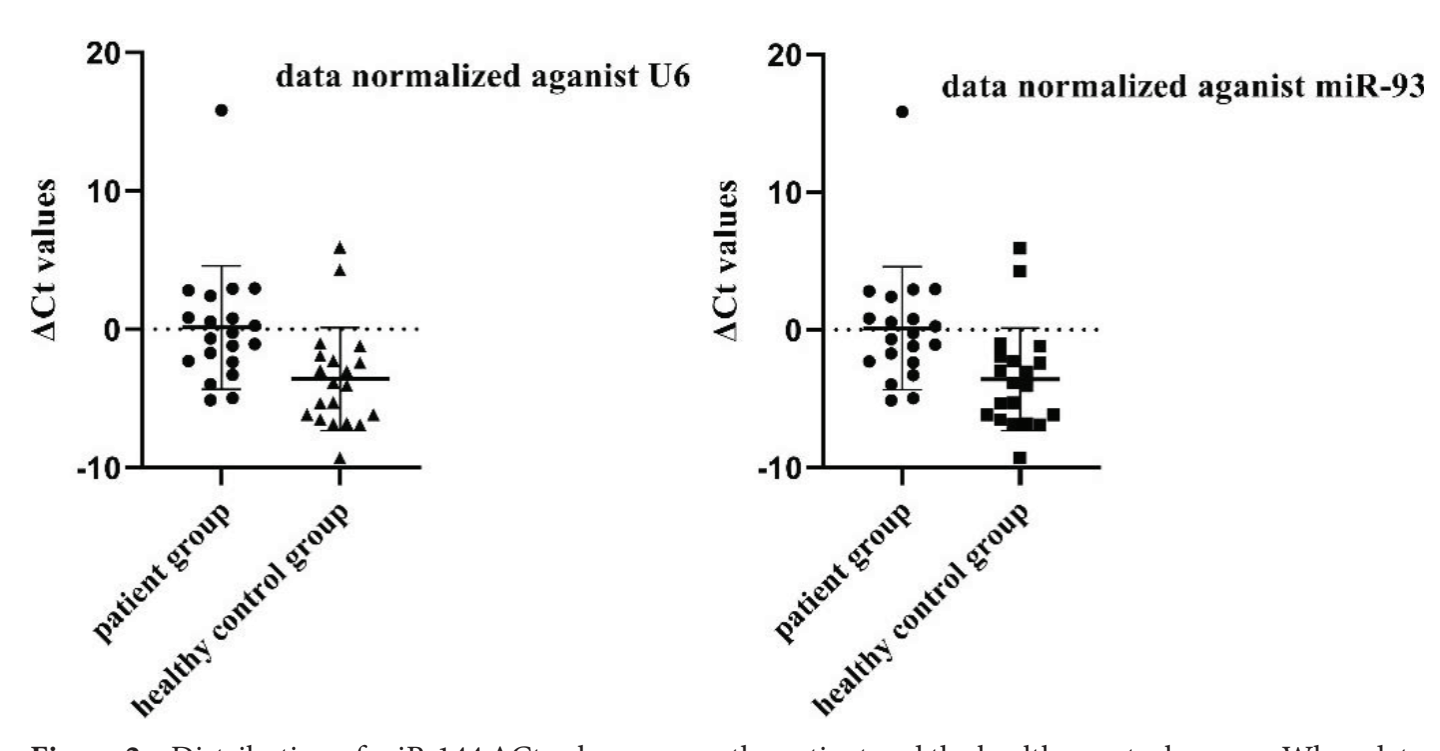
Distribution of miR-144 ∆Ct values among the patient and the healthy control groups. When data were normalized against U6, STD values for the healthy control and the patient groups were 2.88 and 3.76, respectively. When data were normalized against miR-93, STD values for the healthy control and the patient groups were 4.45 and 3.73, respectively.

**Figure 2b:**
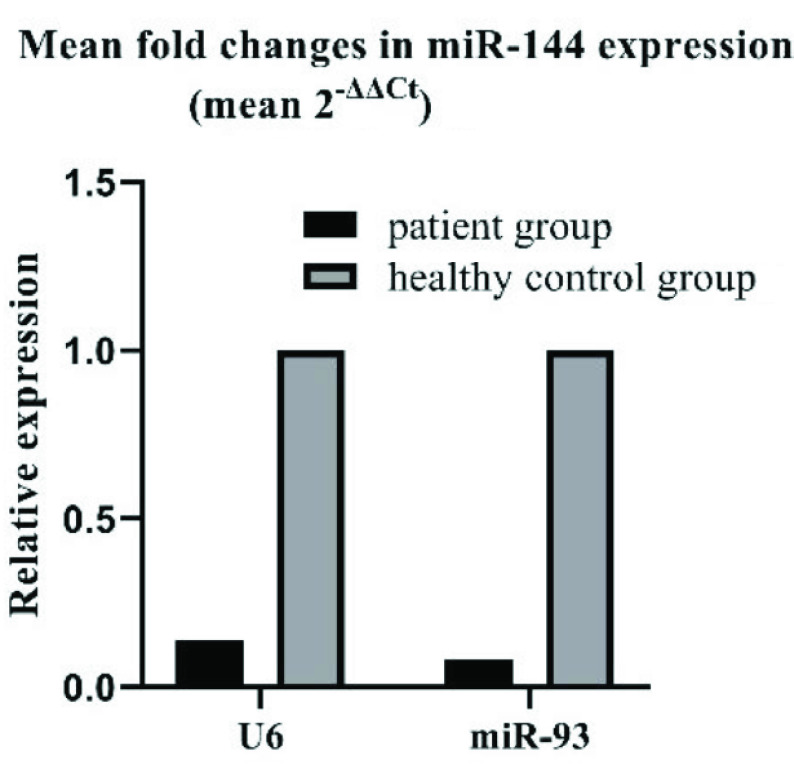
Distribution of miR-144 ∆Ct values among the patient and the healthy control groups. When data were normalized against U6, STD values for the healthy control and the patient groups were 2.88 and 3.76, respectively. When data were normalized against miR-93, STD values for the healthy control and the patient groups were 4.45 and 3.73, respectively.

When the levels of the target miRNAs in serum were normalized by miR-93, statistically significant changes were detected in the expression levels of miR-144 (–0.9, p < 0.05), miR-125b (–0.8, p < 0.05) and miR-146a (–0.7, p < 0.05). No significant difference was detected in the expression levels of other miRNAs (Table 5).

**Table 5:** Fold change distributions in miRNA expression levels when data normalized against miR-93 (*p: 0.033, p: 0.03, p: 0.002).

miRNAs	Fold changes in patients with active pulmonary TB compared to healthy controls
miR-29a	–0.62
miR-21	–0.57
miR-146a	–0.79*
miR-223	–0.40
miR-142	–0.57
miR-125b	–0.83
miR-144	–0.92
miR-155	–0.53
miR-99b	–0.60
miR-361-5p	–0.36
miR-582-5p	–0.54

However, when the expression data were normalized by both U6 and miR-93 only the expression level of miR-144 was found to be statistically significant (–0.8; p < 0.05 and –0.9; p < 0.05 respectively) (Figure 2b). Hence, it was decided that only miR-144 was suitable for further analysis.

In the next step, the diagnostic potential of miR-144 was evaluated by ROC analysis. In order to test the potential of both references, we also compared the results obtained from normalization by U6 or miR-93 separately. Our findings demonstrated that miR-144 could distinguish active pulmonary TB from controls with an AUC (the area under the curve) of 0.72 (95 % CI 0.560–0.880, p:0.017) (Figure 3), based on data obtained from normalization with U6. Similarly, when data from miR-93 normalization was used for ROC analysis, miR-144 could discriminate active pulmonary TB from controls with an AUC of 0.79 for miR-144 (95 % CI 0.644–0.936, p: 0.002) (Figure 3). Suggesting that miR-144 can be a candidate for discriminating the active TB group from the healthy controls.

Moreover, we also determined the diagnostic potential of miR-146a and miR-125b by ROC analysis. Our findings suggested that miR-146a distinguish active pulmonary TB from healthy controls with an AUC of 0.69 (95 % CI 0.530–0.865, p:0.033) and miR-125b distinguish active pulmonary TB from healthy controls with an AUC of 0.70 (95 % 0.533–0.867, p: 0.03) based on data provided from normalization with miR-93.

## 4. Discussion

Tuberculosis is a global health problem with high rates of mortality and morbidity. WHO reported that TB is one of the top causes of death worldwide^3^World Health Organization (2019). Global Tuberculosis Report [online]. Website https://www.who.int/tb/publications/global_report/en/ [accessed 21 July 2020]. . Rapid and accurate diagnosis of TB is important to prevent the spread of this disease. However, molecular diagnostic techniques (PCR, Western Blot, microarray, etc.) are only available in a small number of laboratories. Due to the limited access to the molecular diagnostic tools easier methods that allow for detection of stably expressed novel biomarkers are required for diagnosis and prognosis of TB [1–3].

Detection of stable miRNAs in bodily fluids has opened new avenues for their usage as biomarkers for a number of diseases including TB [7,8,12–34,43,44]. Recent studies also demonstrated that miRNAs can have immune-modulatory roles. For instance, a number of miRNAs were shown to be involved in host-pathogen interactions and inflammatory responses [6,7,9,35–37]. Tuberculosis is defined as granulomatous inflammation which involves inflammatory cells and various host factors. These inflammatory cells can secrete miRNAs into the serum [6,12,16] and recent findings suggest that these circulating miRNAs can serve as useful clinical biomarkers [9,12–34]. A number of different miRNAs are reported to be associated with TB infections and some of the most important ones are summarized in Table 2. Despite a wealth of information on this subject, the association between the changes of serum miRNA levels during TB has not been investigated in Turkish patients so far.

Selection of proper references for normalization is crucial for accurate analysis of qPCR data. U6 RNA is the widely accepted reference for normalization of miRNA expression levels [14,18,31,42]. However, U6 expression levels have also shown to vary depending on the types of diseases, technical variations or personal characteristics [39,40]. MiR-93 has been recently suggested as an alternative reference RNA by Barry et al. [42]. However, some studies also reported conflicting results for miR-93 [14]. The discrepancies may originate from the procedures used for obtaining, handling and storing the specimens. Some other studies also suggest variations in miRNA levels based on ethnical background [16,42,45,46]. In this sense, to be able to get more reliable and accurate results, we have also included miR-93 for data normalization in addition to U6 RNA. 

Firstly, we analyzed our findings by normalizing data against the widely used U6 RNA levels. In accordance with the previous reports [14,16,21,31], we found that serum levels of miR-144 were significantly down-regulated (–0.8 fold, p < 0.05) in active TB patients when compared to healthy group (Table 4). However, despite the obvious decreases in the mean expression levels of miR-29, miR-21, miR-146a, miR-142, miR-125b, miR-155, miR-99 and miR-582-5p in TB patients, these changes were not found to be statistically significant. Normalization of data against miR-93, yielded significant changes in the expression levels of miR-144 (–0.9, p < 0.05), miR-125b (–0.8, p < 0.05) and miR-146a (–0.7, p < 0.05) (Table 5). When we normalized the expression data both for U6 and miR-93, only the expression level of miR-144 was found to be statistically significant (–0.8; p < 0.05 and –0.9; p < 0.05 respectively) (Table 4 and Table 5). In agreement with the previous observations by different research groups, miR-144 levels were frequently reported to be altered in TB patients (Table 2). For instance, miR-144 was previously shown to be expressed in T cells and this miRNA is proposed as a potential biomarker candidate for TB diagnosis [13,17,25].

In general, despite the slight discrepancies, miRNA expression patterns were found to be very similar when normalization was made either against U6 or miR-93 (Table 4 and Table 5). Thus, usage of both U6 and miR-93 as two different references seems to be a suitable and reliable approach for data normalization of miRNA expression. Accordingly, Barry et al. and Barry et al. suggested that miR-93 could be used as an alternative reference miRNA for data normalization [18,42].

The receiver operating curve (ROC) is a tool which can be employed for testing the performance and potential of biomarker candidates. Hence, we assessed the diagnostic potential of miR-144 by ROC analysis. Since both U6 and miR-93 were used for normalization, we also compared the ROC data separately for these two different references. Based on data obtained from normalization with U6, we showed that miR-144 could distinguish active pulmonary TB from healthy participants with an AUC (the area under the curve) of 0.72 (95 % CI 0.560–0.880, p: 0.017). Similarly, when data from miR-93 normalization was used for ROC analysis, miR-144 could discriminate active pulmonary TB from healthy controls with an AUC of 0.79 for miR-144 (95 % CI 0.644–0.936, p: 0.002) (Figure 3). In agreement with previous studies [13,17,25], our findings suggest that miR-144 can hold the potential to be a candidate for discriminating the active TB group from the healthy participants.

As mentioned earlier, in addition to miR-144, the expression levels of miR-125b and miR-146a were also altered in TB patients when the normalization was performed with miR-93 (Table 5). Some researchers pointed that miR-144 has a potential as a biomarker for TB [13,17,25], it was reported that miR-125b may be used for identifying TB [13,14,31,33]. In addition to these results, other reports have concluded that miR-146a is related TB pathologies [14,16,18,21] consistent with these results. It appears that miRNA expression levels differ greatly according to both ethnic groups and the clinical samples examined [16,42,45,46]. For this reason, it is necessary to determine the miRNA variability on the basis of ethnicity and confirm clinical samples which are suitable for TB diagnosis. 

In addition to these results, we sought to get a preliminary view of the possible pathways that these miRNAs might be involved. In this sense, we performed a target gene prediction analysis using online bioinformatics tools^,^ 4TarBase v7.0-DIANA TOOLS (2020). [online]. Website http://diana.imis.athena-innovation.gr/DianaTools/index.php. [accessed 21 July 2020].^5^miRDB (2020). MicroRNA target prediction database [online]. Website http://www.mirdb.org/cgi-bin/search.cgi [accessed 09 November 2020]..

In agreement with the previous reports [13,25], our analysis also revealed that genes (mRNAs) involved in toll-like receptor signaling pathway, Jak-STAT signaling pathway, NOD-like receptor signaling pathway, apoptosis, and pro-inflammatory cytokines might have been among the high-priority targets for these miRNAs (Figure 4). Unfortunately, we were not able to experimentally validate the changes in these target pathways.

In conclusion, we found that the expression level of miR-144 was lower in serum samples of TB patients when compared to healthy controls. In contrast, Liu et al., reported that miR-144* was up-regulated in T cells of active TB patients compared with healthy controls [25]. Similarly, Lv et al. showed that miR-144 levels of TB patients in sputum and serum samples were higher than healthy controls when measured before treatment for TB. They also reported that sputum and serum miR-144 levels were significantly lower in TB patients after treatment [17]. In addition, Spinelli et al. reported that the expression levels of miR-144 were altered in PBMCs and pleural fluid mononuclear cells [21]. However, we could not confirm an increase in the expression of miR-144 as suggested by Lv et al. [17], Liu et al. [25]. In general, our observations were in line with the previous reports [13,17,25]. The changes in the miR-144 expression levels seem to have a potential for TB diagnosis. Since there is no data available on the changes in miRNA profiles of TB patients from Turkey, we could not compare our findings with findings from the Turkish population. 

In this sense, ethnicity can also play a role in these discrepancies. However, it will not be possible to draw accurate conclusions with data obtained from small sample size. We are currently trying to expand our findings in a larger sample size where additional control groups for distinct infectious diseases, noninfectious lung diseases and healthy controls that are free of latent TB infection are included. These points are also the limitations of our study. Today, it is still not clearly understood which underlying mechanisms are involved in transition from latent TB to active TB. Some authors reported that active and latent TB could affect similar pathways. At this point, the detection of which miRNAs are different for these diseases is important for clarifying accurate marker(s) for TB. Several studies of miRNA expression levels in serum/plasma and blood cells have been performed that included subjects with active, latent TB, and healthy controls [19,23,27,28]. Fu et al., Ndzi et al., and Wang et al. reported similar miRNA expression trends for miR-29 and miR-451 which were observed in both active and latent TB groups compared to the controls [19,23,27]. Furthermore, miR-29a, miR-29b, miR-29c, miR- 451, miR-340, miR-424, miR-361, miR-365, miR-155, miR-196-5p, miR-144, miR-223, and miR-21 were reported to be upregulated in active TB compared to latent TB group [19,23,27,28] while miR-150-5p and miR-4292 were found to be down-regulated [19,23,27,28]. Therefore, some miRNAs were described to be important for discriminating active TB and latent TB. However, sample size and the use of additional control groups seem to be the limiting factors for validation of these markers.

We believe that a whole miRNA profiling study should also be necessary for determining novel miRNA candidates as well as validation of the targets at the mRNA or protein level. 

 More importantly, comparison of changes before and after treatment can also reveal valuable information.

Despite the limitations discussed above, this is the first preliminary study in Turkey which investigated the changes in the miRNA levels in TB patients. We found that miR-144 can be a suitable candidate for its’ further evaluation as a potential biomarker for TB. We also showed that U6 and miR-93 can be used in combination as reference for normalization of serum miRNA expression data. 

## Funding

This study was funded by Istanbul University with project number, 24335. 

## Informed consent

Ethical approval was obtained by the Clinical Research Ethics Board of Yedikule Chest Diseases and Thoracic Surgery Training and Research Hospital, Istanbul (Date of approval: 23.11.2016, Protocol No: 2016/60).
